# Epidemiologic Analysis of Alcohol and Tobacco Use: Patterns of Co-Occurring Consumption and Dependence in the United States

**Published:** 2000

**Authors:** James C. Anthony, Fernando Echeagaray-Wagner

**Affiliations:** James C. Anthony, Ph.D., is a professor and Fernando Echeagaray-Wagner, Sc.D., is a former postdoctoral fellow at Johns Hopkins University, School of Hygiene and Public Health, Baltimore, Maryland

**Keywords:** AOD (alcohol or other drug) consumption, smoking, tobacco in any form, epidemiology, prevalence, comorbidity, AOD use pattern, gender differences, age differences

## Abstract

Epidemiologists have conducted nationwide surveys, such as the National Household Survey on Drug Abuse (NHSDA) and the National Comorbidity Survey (NCS), to estimate the prevalence of either the individual or the concurrent consumption of and dependence on alcohol and tobacco. These estimates indicated that for both alcohol and tobacco, use was already relatively high among the youngest respondents, peaked among young adults, and declined in older age groups. A similar pattern existed for concurrent alcohol and tobacco use. Moreover, these estimates showed only moderate gender differences. With respect to dependence, the age-related prevalence patterns differed somewhat for alcohol and tobacco, with the prevalence of tobacco dependence relatively lower among the youngest respondents compared with the prevalence of alcohol dependence. The age-related pattern for concurrent alcohol and tobacco dependence was similar to that found for tobacco dependence.

The concurrent use of alcohol and tobacco and its consequences can be analyzed at numerous levels. For example, basic research has explored the mechanisms through which alcohol and nicotine affect the body—particularly the brain—and lead to dependence. Such investigations have found that both drugs act on the brain through overlapping signaling pathways in which the brain chemical (i.e., neu-rotransmitter) dopamine plays a central role. Moreover, such studies have identified certain protein molecules located on various brain cells (i.e., nicotinic receptors) that interact with nicotine and which, at least indirectly, mediate the reinforcing functions of both alcohol and nicotine. (For more information on the neurotransmitter systems involved in mediating the effects of alcohol and tobacco, see the article in this issue by Little, pp. 215–224.)

Other investigators have examined the breakdown (i.e., metabolism) of alcohol and nicotine, noting an overlap between the metabolic systems that dispose of both drugs in the body. Clinical studies have addressed the effects of both drugs on tissues and organs. Such analyses have found that concurrent alcohol and tobacco use significantly enhances the risk of certain cancers, particularly of the oral cavity (National Institute on Alcohol Abuse and Alcoholism 1998) and can adversely affect cardiovascular health.

Other areas of research aim to elucidate the reasons and mechanisms underlying both alcohol and tobacco use. For example, ethnographic studies have investigated the role of social customs or habits as determinants of concurrent alcohol and tobacco use. Behavioral analyses have focused on the initiation of alcohol and tobacco use that often occurs during adolescence, when young people experiment with multiple adult social roles. According to these studies, adolescents typically begin using such alcoholic beverages as beer and wine, followed by either distilled spirits or cigarettes. Moreover, experimental evidence indicates that alcohol consumption promotes smoking and that smoking possibly promotes drinking. Finally, clinical research on twins has substantiated the idea that common genetic factors may underlie a predisposition to both alcohol and nicotine dependence, and additional research in both humans and animal models is exploring the interplay of environmental and genetic risk factors. (For more information on these genetic and environmental factors, see the article in this issue by Madden and colleagues, pp. 209–214.)

All of these biological and behavioral observations, which focus on the individual, strongly suggest that a causal relationship between alcohol and tobacco use also exists at the higher level of population analysis. Thus, alcohol use may influence tobacco use (and vice versa), not only in the individual but also within a community. To elucidate these population-level influences, however, researchers must determine the extent of alcohol use, tobacco use, and concurrent use of both drugs in the population. This article addresses these epidemio-logical issues using data obtained from two U.S. studies, the National Household Survey on Drug Abuse (NHSDA) and the National Comorbidity Survey (NCS).^1^ The article also examines whether substantial variations in alcohol and tobacco use exist between population subgroups (e.g., different age groups and groups segregated by gender).

## Survey Design

The NHSDA has been conducted annually since the late 1980s, whereas the NCS was conducted in 1990 to 1992. Both surveys are designed and conducted to allow researchers to recruit a representative and valid sample of respondents and to measure the frequency of illicit drug use and other sensitive behaviors. For example, in both surveys the respondents are asked to complete a private, confidential assessment, either marking their responses on an answer sheet that will be seen only by the respondent (in the NHSDA) or providing their responses to an interviewer (in the NCS). (The methodological details of both surveys are described in this article’s sidebar, pp. 203–204.) Moreover, the statistical analyses conducted to yield the resulting estimates of certain behaviors take into account numerous aspects of the survey design and use appropriate estimation procedures. The strengths of these epidemiological survey methods also ensure an accurate assessment of the separate and concurrent use of two licit drugs, alcohol and tobacco.

**Survey Methods**

***The National Household Survey on Drug Abuse***

The National Household Survey on Drug Abuse (NHSDA) is currently conducted annually by the Research Triangle Institute under contract with the United States Substance Abuse and Mental Health Services Administration (SAMHSA), Office of Applied Studies. The survey methods used have been described in detail in each year’s published report and in the scientific literature. In brief, the investigators in recent years have used a complex sampling procedure, called a multistage area probability sampling procedure, to draw each year’s sample of noninstitutionalized U.S. residents age 12 and older. This procedure generates a representative sample, but also allows for oversampling of some population subgroups (e.g., Hispanics and African-Americans) in order to yield a sufficient number of respondents from those groups for valid statistical analyses. This complex survey design necessitates attention to variation in sample selection probabilities and to survey design effects.

Of the designated survey respondents, approximately 75 to 82 percent typically agree to participate. The estimates derived from the survey are statistically modified (i.e., weighted) using certain procedures that help take the varying sampling weights and the nonrespondents into account. These procedures generally assume that people who do not respond to the survey are similar in their characteristics (e.g., drinking and smoking behaviors) to the people who respond. This assumption, however, may not always be correct.

The NHSDA methods used for eliciting self-reports about licit and illicit drug use have been designed to promote self-disclosure of potentially sensitive and illegal behaviors, such as the use of controlled substances (e.g., heroin and cocaine). For example, the respondents mark their answers on special answer sheets that are not seen by the interviewers. In recent years, the survey has introduced an audio, computer-assisted self-interview procedure in which the respondents listen to the questions via audio headphones and reply by entering the response on a laptop computer.

Following the data collection, an initial public report is released and the data are prepared to ensure adequate levels of respondent confidentiality when they are analyzed. Subsequently (i.e., typically 2 to 3 years after the data have been gathered) a public-use data file is released and made available to researchers for analysis. Throughout the preparation of the public-use data files, standard procedures for survey quality control and quality assurance are implemented. Moreover, additional information (e.g., primary sampling unit and strata designators) is supplied with the public-use data files to aid in the statistical analysis.

At the time of preparation of analyses for this article, data from the 1995 to 1997 surveys were available in the public-use data files. The sample sizes for these years were 17,747 respondents in 1995; 18,269 respondents in 1996; and 24,505 respondents in 1997. In this article, the authors chose to show estimates from all 3 survey years to display year-to-year variation. The figures display point estimates from each survey, based on a generalized linear model and smoothing functions designed to generate less variable patterns across age groups.^1^

***The National Comorbidity Survey (NCS)***

The University of Michigan Institute of Survey Research conducted the NCS with primary sponsorship by the National Institute of Mental Health and supplementation by the National Institute on Drug Abuse. Numerous scientific articles have described the NCS survey procedures in detail (e.g., Anthony et al. 1994; Kessler et al. 1994; Warner et al. 1995).

As with the NHSDA, the NCS survey procedures were designed to draw a nationally representative sample of noninstitutionalized U.S. residents, although with a somewhat narrower age range. Thus, the NCS included 8,098 respondents ages 15 to 54. The NCS used the NHSDA procedures to elicit self-reports about alcohol and other drug use with the addition of a special supplement to elicit reports on daily tobacco use and tobacco dependence. The investigators then used standardized self-report diagnostic procedures to assign respondents to one or more categories of mental disorders as defined in the American Psychiatric Association’s (1987) *Diagnostic and Statistical Manual of Mental Disorders, Third Edition, Revised* (DSM–III–R). To generate weighted estimates and 95-percent confidence intervals, the researchers used variance estimation procedures that thoroughly considered the survey sample selection probabilities and complex survey design. For clarity, however, the figures in this article display point estimates.

The NCS did not estimate the number of tobacco smokers. Therefore, NHSDA estimates of the number of tobacco smokers have been used to prepare the figures in this article (see Anthony et al. 1994).

—James C. Anthony and Fernando Echeagaray-Wagner

1Here, point estimates are prevalence proportions. A generalized linear model is a statistical procedure that expresses these proportions as a function of age.

ReferencesAmerican Psychiatric AssociationDiagnostic and Statistical Manual of Mental Disorders, Third Edition, RevisedWashington, DCthe Association1987AnthonyJCWarnerLAKesslerRCComparative epidemiology of dependence on tobacco, alcohol, controlled substances, and inhalants: Basic findings from the National Comorbidity SurveyExperimental and Clinical Psychopharmacology22442681994KesslerRCMcGonagleKAZhaoSLifetime and 12-month prevalence of DSM–III–R psychiatric disorders in the United States. Results from the National Comorbidity SurveyArchives in General Psychiatry511819199410.1001/archpsyc.1994.039500100080028279933WarnerLAKesslerRCHughesMAnthonyJCNelsonCBPrevalence and correlates of drug use and dependence in the United States. Results from the National Comorbidity SurveyArchives in General Psychiatry523219229199510.1001/archpsyc.1995.039501500510107872850

The data presented in this article are based on the NHSDAs conducted from 1995 to 1997 and on the NCS conducted during a fieldwork interval from 1990 to 1992, the most recent years for which data were available for both surveys. In the corresponding figures, the NHSDA-based estimates are presented for each of the 3 survey years to provide a sense of the degree of consistency of these estimates.

For both the NHSDA and the NCS, the respondents were classified according to their ages as measured by their self-reports. The NHSDA survey included respondents age 12 and older, whereas the NCS included respondents ages 15 to 54. To provide more stable estimates of alcohol and tobacco use across various age groups, the statistical analyses of the NHSDA data included a “smoothing function,” a statistical approach that produces a less variable pattern of findings across different age groups by “borrowing” information from adjacent age groups. To quantify the potential burden of alcohol and tobacco dependence in the U.S. population, some new analyses of NCS data have also been conducted.

## Prevalence of Recent Alcohol and Tobacco Use

**Figure 1:**
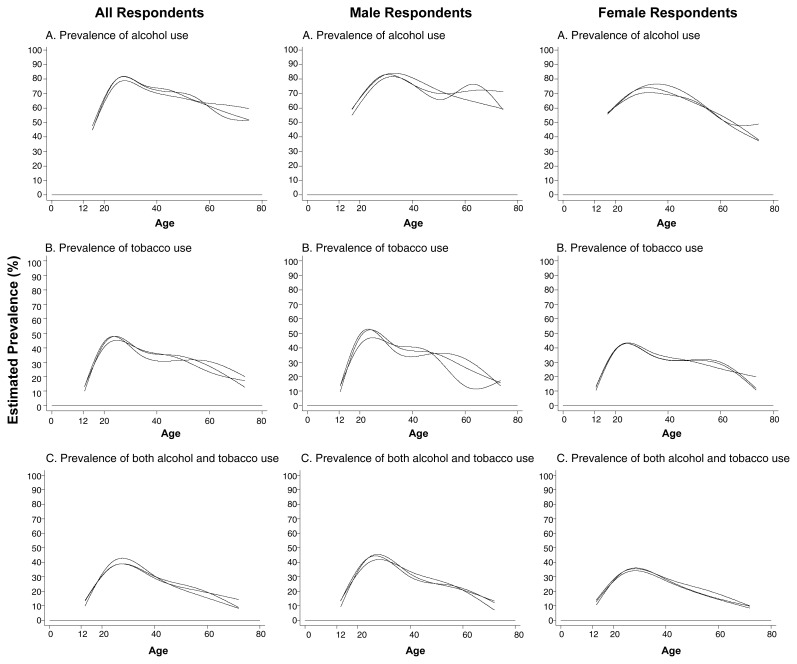
Estimated age-specific and sex-specific prevalence of alcohol and tobacco use during the year preceding each survey. The data were obtained from the National Household Survey on Drug Abuse conducted from 1995 to 1997, with new analyses prepared for this article. Sample sizes were as follows: 17,747 in 1995, 18,269 in 1996, and 24,505 in 1997.

Researchers used the NHSDA data gathered between 1995 and 1997 to estimate the prevalence of recent (i.e., on at least one occasion during the year before the survey interview) use of alcohol and/or tobacco (Branson et al. 1996,^1997^; Substance Abuse and Mental Health Services Administration 1998). The researchers analyzed the data for all age groups, either for all respondents or separately by gender (see figure 1).^2^

### Alcohol Use

With respect to alcohol use (see top panels in figure 1), the youngest respondents (i.e., adolescents ages 12 to 17) already showed a relatively high prevalence of recent use. Thus, 45 to 55 percent of these adolescents had consumed alcohol at least once during the past year. The prevalence of alcohol use was highest among young adults ages 25 to 34, of whom approximately 80 percent had used alcohol during the past year. Among respondents age 35 and older, the prevalence of alcohol use generally declined in a linear fashion with increasing age. In 1 of the 3 survey years, however, an intriguing increase in alcohol consumption was noted in men ages 55 to approximately 70. Although this “uptick” in the curve may simply reflect some statistical instability, the variation may merit a more detailed investigation (e.g., to assess its potential association with retirement or bereavement). Overall, however, alcohol use prevalence among the oldest respondents was similar to that observed during late adolescence. Moreover, with the exception noted here, differences in the prevalence of alcohol consumption between men and women generally were moderate.

### Tobacco Use

The NHSDA similarly estimated the prevalence of recent tobacco smoking in various age groups of the population (see figure 1, middle-row panels). These estimates show a pattern similar to that noted for recent alcohol consumption. Thus, prevalence rates were lowest among young adolescents (i.e., approximately 10 percent among 12-year-olds) but increased sharply during the adolescent and young-adult years, reaching peak values (i.e., approximately 45 to 50 percent) among young adults. Subsequently, a generally linear decline in smoking prevalence occurred among respondents from approximately age 30 to the oldest age groups. As a result, the oldest survey respondents were just as likely as teenagers were to have smoked tobacco in the past year.

Only moderate differences in smoking prevalence existed between male and female survey respondents. The patterns of age-specific prevalence rates also generally were congruent for both genders.

### Concurrent Alcohol and Tobacco Use

The NHSDA allowed researchers to estimate the numbers of people in the U.S. population who had consumed both alcohol and tobacco during the year before the survey was conducted (see bottom panels in figure 1). The analyses found that among the youngest and oldest respondents, approximately 10 to 15 percent of both men and women had consumed both alcohol and tobacco within the past year. Among the young adults, the prevalence of recent concurrent alcohol and tobacco use was approximately 35 to 45 percent. These estimates and their age-specific patterns indicate that a majority of the recent tobacco smokers had also consumed alcohol, whereas the proportion of recent alcohol consumers who had also smoked tobacco recently was smaller.

## Prevalence of Alcohol and Tobacco Dependence^3^

### Dependence Rates Among Alcohol and Tobacco Users

**Figure 2:**
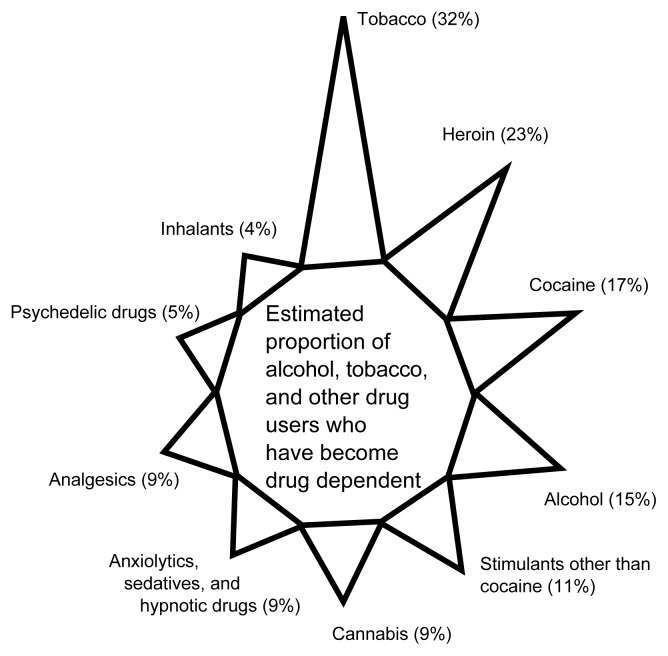
Estimated proportion of alcohol, tobacco, and other drug users who have developed clinical syndromes of drug dependence as defined according to the American Psychiatric Association’s *Diagnostic and Statistical Manual of Mental Disorders, Third Edition, Revised*. The data were obtained from the National Comorbidity Survey, 1990–1992. SOURCE: Adapted from Anthony et al. 1994.

For some people, initial occasional consumption of alcohol and/or tobacco leads to the development of the clinical syndromes of alcohol and/or tobacco dependence. For example, according to the NCS estimates for 15- to 54-year-old U.S. residents, approximately 15 percent of alcohol users had become alcohol dependent and approximately 32 percent of tobacco users had become tobacco dependent (see figure 2) (Anthony et al. 1994).

**Figure 3:**
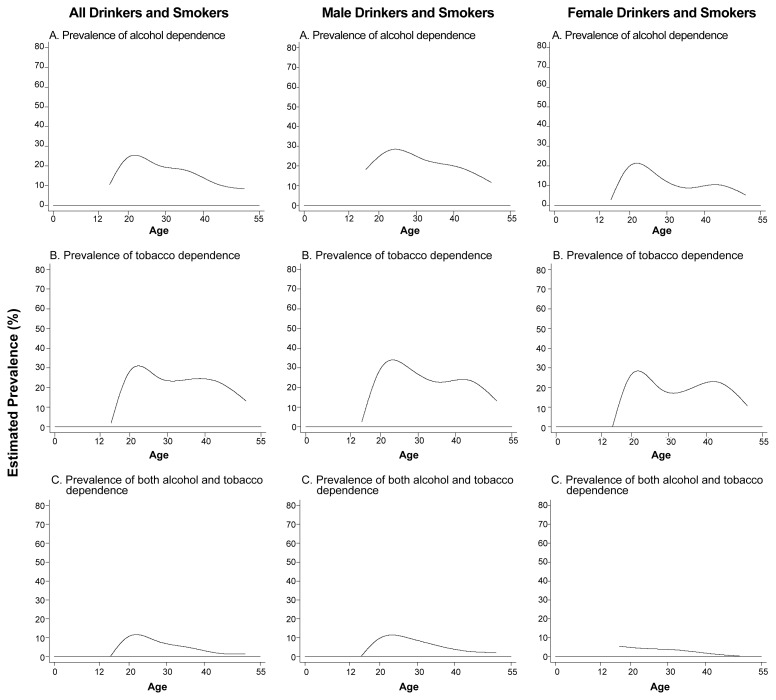
Estimated age-specific and sex-specific prevalence of clinical syndromes of dependence on alcohol and tobacco among recently active users of these drugs, with dependence defined according to the American Psychiatric Association’s *Diagnostic and Statistical Manual of Mental Disorders, Third Edition, Revised.* The data were obtained from the National Comorbidity Survey, 1990–1992, with new analyses conducted for this article.

In order to examine the salience of alcohol and tobacco dependence as determinants of continued alcohol and tobacco consumption among the population, epidemiologists have analyzed data from the NCS to estimate the proportion of recent alcohol and tobacco users who were alcohol and tobacco dependent (see figure 3).^4^ For these analyses, the NCS investigators defined dependence according to the criteria of the American Psychiatric Association’s (1987) *Diagnostic and Statistical Manual of Mental Disorders, Third Edition, Revised* (DSM–III–R).

Based on the DSM–III–R criteria, an estimated 10 to 30 percent of recent alcohol consumers ages 15 to 24 were designated as alcohol dependent (see top panels of figure 3). With increasing age, the prevalence of alcohol dependence among recent users declined almost linearly.

The age-related pattern for tobacco dependence differed from the pattern for alcohol dependence (see middle-row panels in figure 3). For example, unlike alcohol dependence, tobacco dependence was lowest among the youngest tobacco smokers. The prevalence of tobacco dependence increased to approximately 25 to 35 percent among young adult smokers and remained high (i.e., approximately 25 percent) before it declined again among the oldest segment of the study population (i.e., people ages 45 to 54).

Finally, the NCS assessed the respondents’ concurrent alcohol and tobacco dependence in the year preceding the survey interview (see bottom panels in figure 3). As with the estimated prevalence rates for tobacco dependence, concurrent alcohol and tobacco dependence appeared to be less prevalent among 15- to 18-year-old users (i.e., approximately 5 percent) than among older adolescent and young adult users (i.e., approximately 10 percent among 21- to 25-year-olds).

The variation in age-specific prevalence of alcohol dependence and tobacco dependence is particularly pronounced among female alcohol and tobacco users (see right panels of figure 3). Thus, alcohol dependence is more prevalent among young-adult female drinkers (i.e., approximately 20 to 25 percent) than among adolescent and older female drinkers (i.e., approximately 10 percent or less). For tobacco, however, women around age 40 who smoke are almost as likely to be tobacco dependent (i.e., approximately 25 percent) as are young adult women. Conversely, adolescent female smokers have lower rates of tobacco dependence (i.e., less than 20 percent).

Concurrent alcohol and tobacco dependence affects approximately 8 to 10 percent of young female smokers, with a subsequent linear decline (see right panel in the bottom row of figure 3). These values are slightly lower than those observed among male alcohol and tobacco users, in whom dual dependence prevalence peaks at approximately 14 percent at ages 22 to 25.

### Dependence Rates in the Entire Population

The NCS data also provided estimates of the prevalence of dependence on alcohol and tobacco in the population at large (i.e., considering both consumers and nonconsumers of both drugs). When all age groups were combined, the NCS data indicated that approximately 14 percent of 15- to 54-year-olds in the United States had become alcohol dependent and approximately 24 percent of people in those age groups had become tobacco dependent (Anthony et al. 1994). Accordingly, if alcohol dependence and tobacco dependence occurred together only by chance, one would expect approximately 3.4 percent of the study population (i.e., 24 percent of 14 percent) to be both alcohol and tobacco dependent. In fact, however, the NCS found the prevalence of concurrent alcohol and tobacco dependence to be approximately twice as high (i.e., 6.9 percent), suggesting a need for research on mechanisms that may contribute to co-dependence on both drugs.

## Limitations of Survey Studies

When discussing epidemiologic survey estimates, such as the ones presented in this article, researchers must consider several study limitations. First and foremost among these limitations is the generally consistent observation that self-reported alcohol and tobacco consumption tends to understate consumption levels when compared with other data sources (e.g., sales-tax revenues and data on production or supply). Second, the estimates for alcohol and tobacco dependence are based on a lay diagnostic survey method, rather than a stringent psychiatric diagnostic evaluation, and use diagnostic criteria that are not perfect in their validity. Consequently, people who are willing to report symptoms of tobacco dependence might also be more likely to disclose information confirming their symptoms of alcohol dependence and vice versa. As a result, the prevalence estimates of concurrent alcohol and tobacco dependence may be overstated compared with the prevalence estimates for dependence on either drug alone. This limitation, also called “shared methods covariation,” merits greater attention in future studies on alcohol and tobacco use and on co-occurring alcohol and tobacco dependence, particularly in light of the ever-growing social disapproval of tobacco smoking.

## Conclusion

Despite the limitations mentioned in the previous section, the epidemiologic evidence regarding the co-occurring use of alcohol and tobacco is consistent with other clinical, biobehavioral, and neuroscientific evidence. Thus, the evidence points toward patterns of co-occurring use and co-occurring dependence syndromes that deserve greater scrutiny in all of these research domains.

The patterns of co-occurring alcohol and tobacco use in the population motivate a continued search for mechanisms underlying both types of drug use, including mechanisms of reciprocal process, in which alcohol use promotes continued smoking and smoking promotes continued drinking. Similarly, the patterns of co-occurring alcohol and tobacco dependence prompt investigations into underlying shared genetic and neurobiological vulnerabilities to both forms of dependence as well as social and experiential processes that result from or lead to sustained consumption of these drugs.

## Footnotes

1 Other surveys also exist that address these issues, including the National Longitudinal Alcohol Epidemiologic Survey (NLAES).

2 The authors of this article conducted these analyses.

3 Laboratory researchers tend to study or refer to nicotine dependence because they can specifically administer nicotine to their subjects. Epidemiologists, however, often study and refer to tobacco dependence because their data refer to people’s use of tobacco products, which contain numerous substances in addition to nicotine that could potentially contribute to dependence development.

4 The authors of this article conducted these analyses.

## References

[b1-arcr-24-4-201] American Psychiatric Association (1987). Diagnostic and Statistical Manual of Mental Disorders, Third Edition, Revised.

[b2-arcr-24-4-201] Anthony JC, Warner LA, Kessler RC (1994). Comparative epidemiology of dependence on tobacco, alcohol, controlled substances, and inhalants: Basic findings from the National Comorbidity Survey. Experimental and Clinical Psychopharmacology.

[b3-arcr-24-4-201] Kessler RC, McGonagle KA, Zhao S (1994). Lifetime and 12-month prevalence of DSM–III–R psychiatric disorders in the United States. Results from the National Comorbidity Survey. Archives in General Psychiatry.

[b4-arcr-24-4-201] Warner LA, Kessler RC, Hughes M, Anthony JC, Nelson CB (1995). Prevalence and correlates of drug use and dependence in the United States. Results from the National Comorbidity Survey. Archives in General Psychiatry.

